# The benefit of antibiotic-combined Mg-hydroxyapatite bone graft substitute over autologous bone for surgical site infection prevention in posterolateral spinal fusion: a retrospective cohort study

**DOI:** 10.1097/MS9.0000000000000728

**Published:** 2023-04-27

**Authors:** Bruno Zanotti, Francesco Muggiolu, Lucio De Maria

**Affiliations:** a Unit of Neurology With Neurosurgical Activity and Stroke Unit, Mantova Hospital, Department of Neuroscience, University of Mantova, Mantova; b Unit of Neurosurgery, Spedali Civili Hospital, Department of Medical and Surgical Specialties, Radiological Sciences and Public Health, University of Brescia, Brescia, Italy

**Keywords:** antibiotic, bone graft substitute, magnesium-hydroxyapatite, prevention, spinal fusion, surgical site infection

## Abstract

Retrospective cohort study.

**Objective:**

The authors’ goal was to clarify whether a bone substitute combined with antibiotics might gain a hold in spinal surgery as a preventive treatment for early infections (EIs).

**Background:**

A relatively infrequent but severe complication in spinal surgery is the occurrence of EIs.

**Methods:**

The authors retrospectively compared a population undergoing posterolateral fusion with Mg-enriched hydroxyapatite paste mixed with 60 mg rifampicin powder, with a matched population treated with autologous bone without antibiotics. A total of 30 patients from 2020 to 2021 were included in our study. We estimated EI’s relative risk and the number needed to treat. Statistical analyses were performed using the R statistical package v3.4.1 (http://www.r-project.org).

**Results:**

No early infections occurred in the population treated with antibiotic-combined bone substitutes, compared with 6.7% of patients treated with autologous bone without antibiotics. The relative risk of EIs was 0.33 (*P=.*49; 95% CI*=*0.01–7.58) and the number needed to treat was 15.

**Conclusions:**

The results support the hypothesis that combining bone substitutes with antibiotics may decrease the risk of EIs and could be a viable option to improve spinal surgery outcomes. However, a larger sample size would be needed to confirm the benefit of rifampicin-combined Mg-enriched hydroxyapatite substitutes over autologous bone for surgical site infection prevention.

**Level of Evidence:**

Level 3.

## Introduction

Highlights

- Early infections are relatively infrequent but severe complications in spinal surgery.
- We compared a population undergoing posterolateral fusion with a bone substitute combined with antibiotics, with a matched population treated with autologous bone without antibiotics.
- No early infections occurred in the population treated with antibiotic-combined bone substitutes, compared with 6.7% of patients treated with autologous bone without antibiotics.
- Combining bone substitutes with antibiotics may decrease the risk of EIs and could be a viable option to improve spinal surgery outcomes.
- A larger sample size would be needed to confirm the benefit of antibiotic-combined bone graft substitutes over autologous bone for early surgical site infection prevention.

Nosocomial surgical site infections (SSIs) have been defined by the Centers for Disease Control and Prevention (CDC) as those infections due to pathogens driven to the surgical site by various manoeuvres despite preventive measures^1^. Nosocomial SSIs generally occur within 30 days from surgery and are named “early infections” (EIs). If implants are left in place, nosocomial SSIs may occur after 30 days and are defined as “late infections” (LIs)^2^. Likewise other surgical procedures, in spinal surgery, the occurrence of infections has been recently remarked by the Italian scientific community as an event that poses a significant danger to the patient, requiring specific interventions and economic expenditure^3^.

The implantation of any material increases the risk of wound and SSIs by reducing the host’s defenses^4,5^. In the presence of prosthetic material or bone substitutes, a low bacterial load is sufficient to cause infection. Therefore, antibiotic prophylaxis is always implemented in these cases^6,7^. However, an evidence-based surgical practice must be added to antibiotic prophylaxis as the other essential component of an effective infection control policy.

Some implanted materials have been regarded safer than others in preventing surgical SSIs^8,9^, although there is ongoing research to find more suitable prostheses. For those infections involving bone structures, targeted antibiotic delivery using a carrier has become an important management option, as it provides high local concentrations with fewer systemic side effects^10,11^. Among the various absorbable antibiotic carriers developed^12,13^, the hydroxyapatite void filler has shown encouraging clinical outcomes^14,15^. Diverse antibiotics have been studied in combination with different carriers. As for the hydroxyapatite, the antibiotics tetracycline, daptomycin, and rifampicin have shown particular properties of accretion to this bone graft substitute, representing a possible instrument for the prevention and control of bone infections and related SSI^16,17^. Therefore, we conducted a retrospective cohort study to clarify whether the use of rifampicin-combined Mg-hydroxyapatite bone graft substitute may be advantageous in preventing SSIs over autologous bone in posterolateral spinal fusion.

## Materials and methods

### Patient selection

In accordance with the 1975 declaration of Helsinki and under Institutional Review Board approval, we retrospectively analyzed data about over 100 consecutive patients requiring posterolateral spinal fusion from January 2020 to December 2021. The study was conducted according to the “Strengthening The Reporting Of Cohort Studies in Surgery (STROCSS)” criteria^18^.

Inclusion criteria were: (1) patients undergoing lumbar posterolateral spinal fusion through the conventional open posterior approach, (2) concomitant cauda and nerve roots decompression through laminectomy and foraminotomy, (3) biological arthrodesis by means of SINTlife Mg-enriched hydroxyapatite paste (Finceramica) mixed with 60 mg rifampicin powder or by autologous bone without antibiotics, (4) age more than 18 years, and (5) follow-up of minimum 1 month. We excluded patients with serious risk factors for SSIs, such as prior surgery in the same area, other sites of infection, diabetes, cardiovascular impairment, malnutrition, immunodeficiencies, or protein deficiencies. Patients responding to inclusion and exclusion criteria were divided depending on the type of biological arthrodesis to obtain two series of patients. Finally, patients were matched among the two series to obtain comparable sex and age.

### Outcomes and endpoints

Postoperative infections were classified as EI, if within 30 days from the operation, and LI, if after 30 days from the surgery^2^. Infection rates were compared between the two cohorts. Cohort A was composed of patients who underwent biological arthrodesis with Mg-enriched hydroxyapatite paste mixed with 60 mg rifampicin powder and cohort B was composed of patients who underwent biological arthrodesis with autologous bone without antibiotics.

Our primary objectives were EI’s relative risk (RR) and the number needed to treat. The secondary endpoints were the EI’s RR reduction and the number needed to harm, the attributable risk and the absolute risk reduction.

### Statistical analysis

Descriptive statistics were reported including ranges and percentages. All statistical analyses were performed using the R statistical package v3.4.1 (http://www.r-project.org). Formal statistical comparisons were performed in spite of small sample sizes and insufficient power to detect differences between groups. The level of statistical significance was set with *P* less than .05.

## Results

### Baseline data

**Figure 1:**
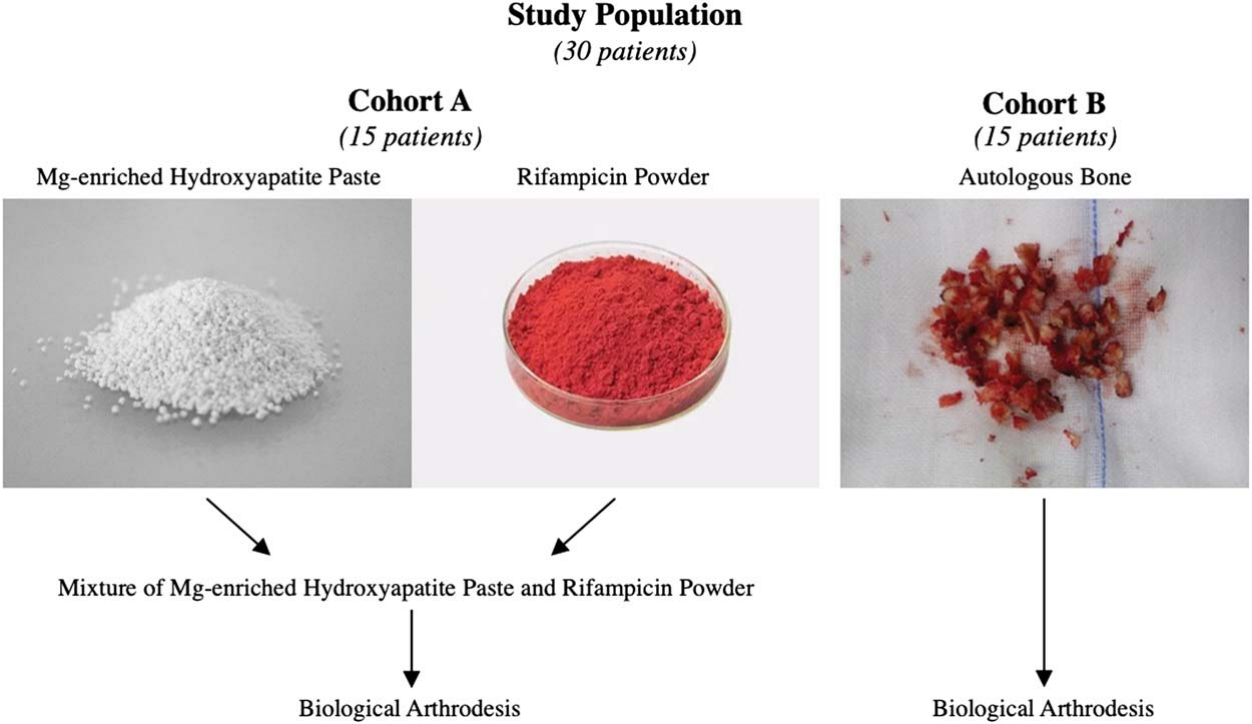
Image showing the difference between the two groups of the study population. Cohort A (left) was composed of 15 patients who underwent biological arthrodesis with Mg-enriched hydroxyapatite paste mixed with 60 mg rifampicin powder. Cohort B (right) was composed of 15 patients who underwent biological arthrodesis with autologous bone prepared from a local bone source during neural decompression.

A total of 30 patients were included in the study (Fig. 1). Cohorts A and B populations were matched to obtain comparable sex and age (Table 1). None of the 30 patients had serious risk factors for SSIs, such as prior surgery in the same area, other sites of infection, diabetes, cardiovascular impairment, malnutrition, immunodeficiencies, or protein deficiencies. Furthermore, all the patients included in the study population underwent standard antibiotic prophylaxis^3,19^.

**Table 1:** Baseline data of the study population

Cohorts	No. patients	No. males / females (%)	Median age (range)	Biological arthrodesis
(A)	15	10 males (66.6%) / 5 females (33.4%)	60.4 years (range 36–79)	Mg-enriched hydroxyapatite paste with 60 mg rifampicin powder
(B)	15	11 males (73.3%) / 4 females (26.7%)	65.8 years (range 35–85)	Autologous bone without antibiotics

### Primary and secondary endpoints

Infection of the surgical site occurred in one patient of cohort B (6.7%) within 30 days from the surgery (EI), and the patient did require reoperation. No patients (0%) developed SSI 30 days after surgery (LI). No patients (0%) of cohort A developed SSIs.

Patients treated with autologous bone without antibiotics had a three times greater risk of developing EI (RR*=*0.33; *P=.*49; 95% CI*=*0.01–7.58). A number of 15 patients treated with antibiotic-combined bone substitutes rather than autologous bone without antibiotics would be needed to prevent EI in 1 patient (number needed to treat *=*15; 95% CI*=*4.37–**∞**). The RR reduction was 0.67 (95% CI −6.58 to 0.99). A number of 14 patients treated with autologous bone without antibiotics rather than antibiotic-combined bone substitutes would be needed for 1 patient to develop EI (number needed to harm = 14; 95% CI*=*9.63–**∞).** The attributable risk was −6.67% (95% CI*=*−23.30 to 9.97%) and the absolute risk reduction was 6.67% (95% CI*=*−9.97 to 23.30). A summary of the results is reported in Table 2.

**Table 2:** Summary of primary and secondary endpoints

	X (%)	95% CI
Primary endpoints		
Relative risk (RR)	0.33	0.01–7.58
Number needed to treat (NNT)	15	4.37–**∞**
Secondary endpoints		
Relative risk reduction (RRR)	0.67	−6.58 to 0.99
Number needed to harm (NNH)	14	9.63–**∞**
Attributable risk (AR)	−6.67%	−23.30 to 9.97%
Absolute risk reduction (ARR)	6.67%	−9.97 to 23.30%

### Illustrative case

**Figure 2:**
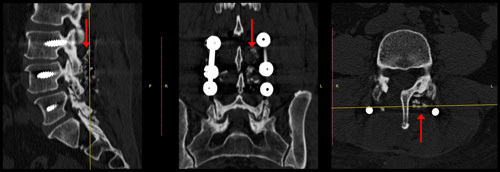
Spinal CT scans the day after surgery. On the left side (red arrows) is the autologous biological reinforcement bone powder for titanium-instrumented arthrodesis. CT, computed tomography.

**Figure 3:**
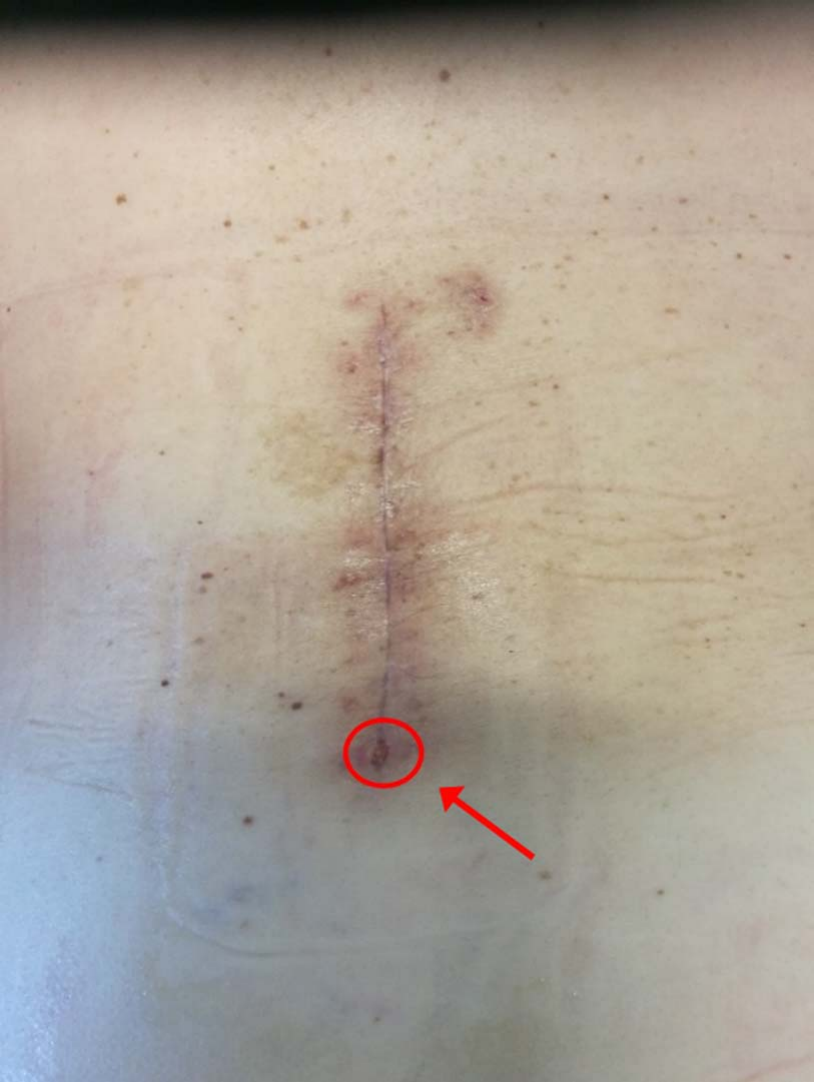
Surgical wound 18 days after surgery. Slightly red and with diastasis in its lower portion (red arrow and circle).

Female 53-years-old patient. No diabetes nor other comorbidities were reported. No known allergies. Recent history of progressive lumbago radiating down to the right lower limb. Neuroradiological tests showed unstable first-degree anterolisthesis of L4 on L5. After standard antibiotic prophylaxis with 2 g of cefazolin, L3-L4-L5 instrumented vertebral arthrodesis with autologous bone powder reinforcement was performed (Fig. 2). There were no perioperative complications. However, 18 days after surgery, the patient had an oozing of serum and corpuscles from the distal end of the wound (Fig. 3). Failure of 6 days of treatment P.o. with 1 g of amoxicillin-clavulanate t.i.d., led to hospitalization for investigations and further treatment. Due to wound infection, the patient was started on antibiotic therapy, first on an empirical basis with i.v. piperacillin-tazobactam 4.5 g t.i.d. for 7 days, and then following antimicrobial susceptibility testing with i.v. vancomycin 20 mg/kg b.i.d. for 6 weeks. Wound swabs detected methicillin-resistant Staphylococcus aureus. Revision surgery of the wound followed. After 6 months, the surgical wound appeared to be healed. Phlogosis indices reduced over time. Vital signs were normal at follow-up.

## Discussion

### General data on spinal SSIs

Although complications in spinal surgery were initially underestimated at 16%^3^, today we are well aware that the percentage is likely to be higher. An American review reports it at as high as 45%^20^, but we do not have clear comparison data for Europe. Among post-surgical infections, that is occurring whenever there is a skin incision, those directly related to the use of medical devices, however, are clinically insignificant. Risk factors include very advanced age, prior surgery in the same area, other infections, diabetes, alcoholism, malnutrition, immunodeficiencies, or protein deficiencies. However, despite the presence of risk factors, early or late SSIs directly related to the use of synthetic materials for stabilization procedures are rare if compared to the overall rate of SSIs^21–23^. Even though we observed one only SSI within 30 days from surgery (EI), it is important to consider that nosocomial SSIs may also occur after 30 days (LI) in posterolateral spinal fusions, as in any surgery where implants are left in place^2^.

The infection risk in surgical procedures is related to the breakdown of our main barrier of defense against the outside world, that is the skin. Infection in spinal surgery is undoubtedly a recurrent and negative event. However, the percentage is quite low, with a frequency of about 1%. Intravenous antibiotic therapy to prevent infection is usually given before surgery, and sometimes even during surgery if it is to last several hours^24–27^. Postoperatively, in high-risk cases, antibiotic treatment may be continued^28,29^. Particular attention is paid to the preparation of the operating area and to the surgical technique performed under maximum sterility.

In spinal surgery, infections can be isolated to the vertebrae (spondylitis), discs (discitis), or both (spondylodiscitis). The incidence has the first weaker peak during the second decade of life (from 10 to 20 years), while the most important peak is mainly found in adults over 50^30^. In paediatric patients, the rich vascularization of the immature disc promotes the onset of discitis (3% of cases, without sex difference)^31,32^. The vascularization of the disc disappears at the age of 20, and therefore in adults, the infection develops primarily in the vertebra, extending only secondarily to the disc, configuring the typical picture of spondylodiscitis^33,34^. Anatomical structures contiguous to the spine, meninges, marrow, nerves, and paravertebral muscles may also be affected to a variety of degrees by the infectious process. The infectious site may be located at all levels of the spine, but most often is in the lumbar region (60–70% of cases)^35–37^.

### Involved pathogens and clinical presentation

The pathogenic micro-organisms responsible for spondylodiscitis vary depending on the mode of contamination (haematogenic in 60–80% of cases, direct inoculation in up to 15%, and by contiguity in 13% of cases), geographical area, age, and predisposing factors, in particular prior surgery and states of immunodepression^38,39^. In the western world, the micro-organisms most responsible for these infections are Gram-positive and Gram-negative pyogenic bacteria, with a clear prevalence for Staphylococcus aureus (up to 50% of cases), followed by Streptococcus, other Staphylococci, Enterobacteria of the type Escherichia Coli, Pseudomonas and Enterococci^40–42^. However, in developing countries, tuberculosis bacillus (Mycobacterium Tuberculosis) prevails, but the incidence of tuberculosis continues to increase worldwide^43^. Infectious pyogenic spondylodiscitis accounts for 2–4% of all cases of osteomyelitis^44^. Fungal spondylodiscitis (Candida, Aspergillus), which is rarer, develops following direct venous inoculation or hematogenous dissemination in subjects at risk (drug use, chronic alcoholism, chemotherapy in diabetic patients)^45^.

The main symptoms of spondylodiscitis are fever, pain, and stiffness of the spine. However, fever is inconsistent and less common in cases caused by the tubercular bacillus^46^. Inflammatory, deep, and constant pain may also be absent in 10% of cases, especially in those of tubercular origin^34^. Contracture of the paravertebral muscles is a more constant symptom and causes decreased mobility of the spine^20^. Neurological, motor and/or sensory deficits, medullary and root compression, with possible sphincter disorders, and involvement of the medullary cone, may occur in advanced cases complicated by vertebral collapses or invasion of the vertebral canal. However, signs and symptoms of systemic infection, such as fatigue, night sweats, and increased inflammation indices, may not be present in all cases^47,48^.

### Italian guidelines for SSI antibiotic prophylaxis

In patients undergoing spinal surgery, standard antibiotic prophylaxis is cefazolin 2 g 30–60 min prior to skin incision, the intraoperative dose is 1 g every two hours after the second hour, or 2 g every 4 h after the fourth hour. If allergies are present, clindamycin is used in the same doses^3^. This protocol is in accordance with the WHO global guidelines for the prevention of surgical site infection ^19^.

### Types of spinal SSIs

As with all surgery, infection in the spinal area may be superficial or deep^49^. Superficial infection involves the skin and the underlying extra-fascial contiguous tissue (dermis). It presents with pain localized around the surgical wound site, with redness, swelling, and heat in the skin around the skin incision, there is often a discharge of yellow fluid, which is sometimes purulent, and usually foul-smelling. As stated above, treatment with oral antibiotics and skin disinfection is usually sufficient for healing^50,51^.

Deep infection involves deep tissues, generally at the level of the spine with possible involvement of the disc, contiguous tissues, spinal instrumentation, if present, and sometimes even vertebral bone tissue. Deep infection presents with pain and often, but not always, fever, chills, night sweats, fatigue, or malaise. This type of infection is very serious and treatment requires intravenous administration of antibiotics over a prolonged period of time, as long as bed rest, and sometimes even early or late reintervention^52–54^.

### Antibiotic-combined instrumentation for spinal SSI prevention

In our population, we observed three times higher risk of developing EIs for patients treated with autologous bone without antibiotics. However, in light of the above, spinal SSIs appear to be more associated with the patient’s general condition. Nevertheless, patient selection was limited to subjects with no comorbidities. Thus, the question remains whether the combination of a synthetic carrier with antibiotics could have avoided this complication. This hypothesis is supported by recent studies reporting a lower risk of SSIs after the use of antibiotic-combined bone grafts for lumbar posterolateral fusion^55,56^. Another study reported lower rates of deep SSIs with the washing of implants and autogenous bone grafts and the irrigation of the surgical field using diluted rifampicin but concluded that larger series are needed to verify these results^57^. Likewise, a larger sample size would be needed to confirm the benefit of rifampicin-combined Mg-enriched hydroxyapatite substitutes over autologous bone for SSI prevention. Furthermore, a cost analysis may be helpful to evaluate the impact of antibiotic-combined substitutes in terms of finite resources.

Many antibiotics have been studied for SSI management^6^. Some of them have shown peculiar aspects that may be suitable for infections potentially involving bone structures. Biomaterials such as hydroxyapatite may act as recruiting moiety for some particulate-seeking drugs^14,58^. The antibiotics tetracycline, daptomycin and rifampicin have been shown to chemically interact with calcium, phosphate, or hydroxyl groups on the hydroxyapatite crystal^17^. For their accretion properties to the hydroxyapatite carrier, these drugs have been increasingly chosen in combination with bone graft substitutes^16,17^. Even though our study focused on the rifampicin-combined Mg-hydroxyapatite bone graft substitute, we believe that future studies should also evaluate the potential of other antibiotics with the same properties in combination with different bone graft substitutes.

### Limitations

Our study has limitations related to the small sample size and consequent lack of statistical power. Larger prospective cohort studies would be needed to reach statistical significance. Moreover, even though we do not believe that a matched cohort of patients undergoing biological arthrodesis with Mg-hydroxyapatite bone graft without antibiotics could have resulted in a different rate of SSI, if compared with patients undergoing arthrodesis with autologous bone, the use of the same bone substitute would have been preferable form a methodological point of view. However, given the retrospective nature of this study, we did not have a comparison cohort of patients who underwent biological arthrodesis with Mg-hydroxyapatite bone graft without antibiotics.

Nonetheless, this is the first study comparing the rate of SSIs in patients undergoing posterolateral spinal fusion with Mg-hydroxyapatite bone combined with rifampicin versus autologous bone without antibiotics. Although not statistically significant, these results support the hypothesis that the combination of bone graft substitutes with antibiotics prevents SSIs and could be a viable option to improve spine surgery outcomes.

## Conclusions

The intraoperative use of Mg-enriched hydroxyapatite paste associated with rifampicin may find a useful application in lumbar posterolateral spinal fusion to prevent the early occurrence of SSIs. Larger studies would be needed to confirm the benefit of rifampicin-combined Mg-enriched hydroxyapatite substitutes over autologous bone for SSI prevention.

## Ethical approval

The study was conducted in accordance with the 1975 declaration of Helsinki and under Institutional Review Board approval.

## Consent

All the patients included in the study provided informed consent. Written informed consent was obtained from the patient for publication of this retrospective cohort study and accompanying images. A copy of the written consent is available for review by the Editor-in-Chief of this journal on request.

## Source of funding

This research did not receive any specific grant from funding agencies in the public, commercial, or not-for-profit sectors.

## Conflicts of interest disclosure

The authors declare they have no financial or other conflict of interests in relation to this research and its publication.

## Provenance and peer review

Not commissioned, externally peer-reviewed.
